# From Fossil to Bio-Based AESO–TiO_2_ Microcomposite for Engineering Applications

**DOI:** 10.3390/polym16233363

**Published:** 2024-11-29

**Authors:** Cristian-Dragos Varganici, Liliana Rosu, Dan Rosu, Mihai Asandulesa

**Affiliations:** 1 Centre of Advanced Research in Bionanoconjugates and Biopolymers, “Petru Poni” Institute of Macromolecular Chemistry, 41A Gr. Ghica–Voda Alley, 700487 Iasi, Romania; lrosu@icmpp.ro (L.R.); drosu@icmpp.ro (D.R.); 2 Department of Electroactive Polymers and Plasmochemistry, “Petru Poni” Institute of Macromolecular Chemistry, 41A Gr. Ghica–Voda Alley, 700487 Iasi, Romania; asandulesa.mihai@icmpp.ro

**Keywords:** epoxy, reactive diluent, vegetable oil, thermal curing, titania, microcomposite

## Abstract

Environmental issues and the reduction of fossil fuel resources will lead to the partial or total substitution of petroleum-based materials with natural, raw, renewable ones. One expanding domain is the obtaining of engineering materials from vegetable oils for sustainable, eco-friendly polymers for different applications. Herein, the authors propose a simplified and green synthesis pathway for a thermally curable, acrylated and epoxidized soybean oil matrix formulation containing only epoxidized soybean oil, acrylic acid, a reactive diluent (5%) and just 0.15 mL of catalyst. The small amount of reactive diluent significantly reduced the initial system viscosity while eliminating the need for adding solvent, hardener, activator, etc. Both the thermally cured composite with a 2% TiO_2_ microparticle filler and its pristine matrix were comparably characterized in terms of structural, thermal, morphological, dielectric and wettability by Fourier transform infrared spectroscopy, differential scanning calorimetry, thermogravimetry, scanning electron microscopy, broadband dielectric spectrometry and contact angle measurements. The 2% filler in the composite generated superior thermal stability via lower mass loss (48.89% vs. 57.14%) and higher degradation temperatures (395 °C vs. 387 °C), increased the glass transition temperature from −20 °C to −10 °C, rendered the microcomposite hydrophobic by increasing the contact angle from 88° to 96° and enhanced dielectric properties compared to the pristine matrix. All investigations recommend the microcomposite for protective coatings, capacitors, sensors and electronic circuits. This study brings new contributions to green chemistry and sustainable materials.

## 1. Introduction

Environmental and economic issues, together with the reduction in petroleum oil reserves, have motivated both academia and industry to seek replacements derived from natural and sustainable resources (lignin, rosin acids, vegetable oils, chitosan, fats) [1,2,3,4,5]. Epoxides are one of the most important classes of resins used in obtaining different thermosets for various application fields. Epoxy resins account for 70% of the commercial markets and have a wide range of applications, from adhesives and coatings to aerospace [6,7]. Cured epoxy matrices exhibit excellent abrasive and chemical resistance and thermal, mechanical and electrical behavior, being well-known as very good insulators [8,9].

However, most epoxy resins are synthesized from the environmentally hazardous petrochemical epoxy monomer diglycidyl ether of bisphenol A (BPA). BPA is an endocrine-disrupting compound that also affects the central and immune system [10,11,12]. Furthermore, a thermosetting formulation may be composed of the epoxy resin, curing agent, reactive diluent, filler, activator, catalyst, etc. Hence, BPA-based epoxies are cured with low molecular weight petroleum-based hardeners, such as amines, anhydrides, polyacids and Lewis acids, that are known to be toxic agents to both human health and the environment [13,14,15,16,17,18]. Moreover, most of these components increase system viscosity. Epoxy resins are known for their high viscosity at room temperature [15,16] and poor toughening [17]; these aspects leading to difficulties in processing and applications that are overcome by solvent addition, of which most are toxic. Reactive diluents may surpass these impediments. Jagtap and More [19] recently reviewed reactive diluents for epoxy resins. Of these, the acrylate-based multifunctional monomer trimethylolpropane trimethacrylate (TMPTMA) significantly enhanced the crosslinking density of epoxy acrylates without adding toxicity.

All the aforementioned aspects have compelled a need to develop sustainable epoxy systems from renewable resources [20] following one of green chemistry’s principles stated by Anastas and Eghbali [21]. Among these resources, during the last two decades, vegetable oils—especially soy-bean and linseed—have received increasing attention due to their availability, low cost, chemical functionality and facile processing [22,23]. Due to their very low reactivity during polymerization, the double bonds in vegetable oils need to be converted into reactive functional groups. Vegetable oils can undergo epoxidation and, when cured with an adequate hardener, produce a bio-based, sustainable epoxy resin [24,25]. Due to its versatility, the epoxide ring can be further converted into a wide palette of functional groups through different reactions, such as with acids (e.g., acrylic), anhydrides or hydroxyls (alcohols, thiols, diols) [26]. Therefore, the development of soy-based epoxy resins for engineering applications remains a challenge for polymer composite industries. Hence, both academic and industrial research is greatly expanding to explore the feasibility of producing polymer composites based on epoxidized soybean oil. Such bio-based compounds may constitute raw materials that can be used to obtain polymeric formulations for micro- and nanocomposites (e.g., with metal oxides) for the protection of different substrates (wood, metal, plastic) [27,28,29,30,31]. Examples include SnO_2_, Al_2_O_3_, Ag or their combined systems (Ag–TiO_2_), which are biocompatible and have excellent antibacterial properties. ZnO nanoparticles offer excellent UV and antibacterial protection. CeO nanoparticles are excellent insulators due to their high chemical resistance. Among all the examples, TiO_2_ is the most widely used due to its relatively small particle size, proven intercalation chemistry, facile processing and many different forms, such as nanowires, nanowhiskers, nanotubes, etc. TiO_2_ also possesses a high chemical stability and surface area—given its availability as nanocrystals and nanodots—as well as attributes like nontoxicity, hydrophilicity, high commercial availability and low cost, among others. TiO_2_ is also commercially available in three metastable phases: rutile, anatase and brookite [32]. TMPTMA may replace traditional curing agents in different materials: (i) macroporous poly(glycidyl methacrylate-co-trimethylolpropane trimethacrylate) with fine-controlled porosity; (ii) poly(hydroxyethyl methacrylate)-based nanocomposites applied in dentistry; (iii) organic columns obtained through controlled or free-radical polymerization that are used as a stationary phase in the technique of capillary liquid chromatography, and (iv) silicone rubber [19].

This is the first report on the synthesis and characterization of a thermo-curable formulation based on epoxidized and acrylated soybean oil (AESO) containing TMPTMA as a reactive diluent. This formulation was used as a matrix to obtain a bio-based composite with a TiO_2_ microparticle filler (AESO–TiO_2_). The scope of this research was: (i) to reduce system viscosity; (ii) to simplify the system and its synthesis towards a green approach by eliminating the use of environmentally and health hazardous solvents and curing agents in the matrix formulation to engineer sustainable microcomposites.

## 2. Experimental Section

### 2.1. Materials

The epoxidized soybean oil used in this study is a product under the commercial name Lankroflex^TM^ E2307 from Valtris Speciality Chemicals (Manchester, UK) (molar mass: 975.399 g mol^−1^; density: 0.997 g mL^−1^; acid value: 0.7 mg KOH g^−1^; viscosity: 325 mPa·s at 25 °C; boiling point: 150 °C at 0.5 kPa; flash point: 310 °C). According to the manufacturer, Lankroflex™ E2307 is a low odor epoxy resin and certified as safe for food contact and medical applications. It is used in all types of PVC formulations in combination with metal soap stabilizers. It is also used as a plasticizer for coating applications, being an excellent water, oil and solvent repellent. Acrylic acid (98% purity) was purchased from Acros Organics, Geel, Belgium and used as received. Methylhydroquinone was a product of Aldrich Chemistry, Wuxi, China. Triethylamine was a product of Sigma–Aldrich, Darmstadt, Germany. Trimethylolpropane trimethacrylate (TMPTMA) was purchased from Sigma–Aldrich, St. Louis, MO, USA and used as received. The TiO_2_ (IV) microparticles (99%) (d = 1–1.3 µm) were purchased from abcr GmbH, Karlsruhe, Germany.

### 2.2. Synthesis of AESO

In a glass flask with a round bottom (500 mL capacity) with three necks, a water cooler and a mechanical stirrer, 100 g (0.102 mol) of epoxidized soybean oil, 34.3 g of acrylic acid (0.465 mol) and 0.04 g (3.2 × 10^−4^ mol) of methylhydroquinone—used as a thermal polymerization inhibitor—were mixed. The temperature required for the reaction was ensured with a silicon oil bath equipped with a thermostat. The reaction mixture was preheated to 40 °C for 30 min, followed by the addition of 0.15 mL of triethylamine. To complete the chemical reaction, mechanical stirring was continued for another 6 h at 70–80 °C. After the completion of the reaction, the AESO was washed a few times in a funnel with saline solution (28 g 100 mL^−1^) to remove the unreacted acrylic acid and dried on anhydrous sodium sulfate. After filtration, 267 g of AESO was obtained with a reaction yield of 98.8%. The final product had yellow-orange coloring and an oily texture. The characterization of AESO by the ^1^H–NMR technique is detailed in the Supplementary Materials (Text S1, Figure S1).

### 2.3. Obtaining the AESO–TiO_2_ Microcomposite

**Figure 1:**
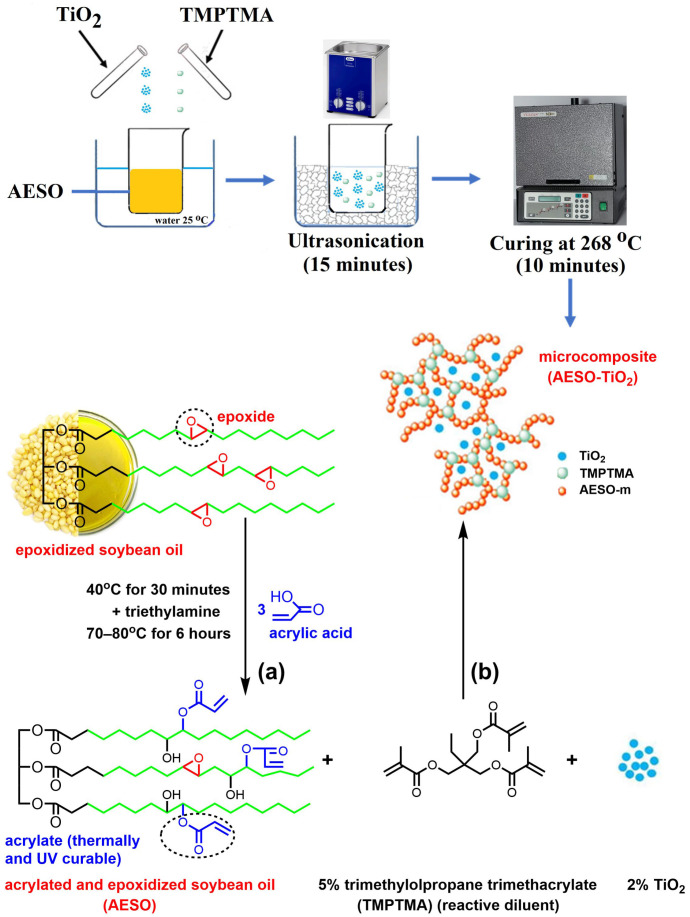
(**a**) Synthesis of AESO and (**b**) obtaining the cured epoxy matrix and microcomposite.

There were stirred together 44.75 g of AESO and 2.521 g (5.63%) of TMPTMA until the homogeneous acrylated epoxy formulation for the matrix (AESO–m) was obtained. Afterwards, the AESO–m formulation was mixed with 0.89 g (2%) of TiO_2_ microparticles in an ultrasonication bath (S 15 Elmasonic from Elma Schimdbauer GmbH, Singen, Germany) for 15 min, and the AESO–TiO_2_ formulation was obtained. The thermally curable AESO–TiO_2_ liquid formulation had a white, pasty texture. In order to obtain the AESO–TiO_2_ composite, the experimental thermal conditions needed to be established. In this sense, 10 mg of the composite formulation was first placed in a pierced and sealed aluminum crucible and its differential scanning calorimetry (DSC) heating curve was recorded from room temperature to 300 °C at a rate of 10 °C min^−1^. A broad, exothermal peak was observed at 268 °C, describing the thermal curing process, as shown in the Supplementary Materials (Figure S2). Then the rest of the composite formulation was heated in an oven (Vulcan 3–130 from Dentsply Ceramco, York, PA, USA) in the same conditions and held for 10 min at 268 °C to obtain the solid white and hard AESO–TiO_2_ microcomposite (Figure 1b).

### 2.4. Methods

#### 2.4.1. Proton Nuclear Magnetic Resonance (^1^H–NMR)

The ^1^H–NMR spectrum of AESO was recorded on an Advance DRX 400 (Brüker, Bremen, Germany) with CDCl_3_ used as a solvent. The device was equipped with a 5 mm direct detection z-gradient probe.

#### 2.4.2. Fourier Transform Infrared Spectroscopy (FT-IR)

The FT-IR spectra were registered on a Vertex 70 apparatus (Brüker, Bremen, Germany) equipped with a MIRacle^TM^ ATR accessory provided with a diamond crystal plate with a 1.8 mm diameter. The liquid formulations were measured within KBr pellets, the solid cured matrix and the composite using the ATR module.

#### 2.4.3. Thermogravimetric Analysis (TGA)

The thermogravimetric curves were recorded on a STA 449 F1 Jupiter apparatus (Netzsch–Gerätebau GmbH, Selb, Germany). Approximately 14 mg of the samples were placed and heated in alumina crucibles. A heating rate of 10 °C min^−1^ was applied in a nitrogen atmosphere (flow rate of 50 mL min^−1^) up to 700 °C.

#### 2.4.4. Differential Scanning Calorimetry (DSC)

The differential scanning calorimetry curves were recorded on a DSC 200 F3 Maia apparatus (Netzsch–Gerätebau GmbH, Selb, Germany). Approximately 10 mg of the samples were placed and heated in aluminum crucibles, which were sealed and shut with pierced lids. Experiments were conducted in a nitrogen atmosphere (flow rate of 50 mL min^−1^) and with a heating rate of 10 °C min^−1^. Calibrations were made with standard indium.

#### 2.4.5. Scanning Electron Microscopy (SEM)

The scanning electron microscopy micrographs were recorded on a Verios G4 UC scanning electron microscope (Thermo Fisher Scientific, Brno-Cernovice, Czech Republic). The samples were fixed onto aluminum stubs using double adhesive carbon tape and, afterwards, coated with 6 nm of platinum with a Leica EM ACE200 sputter coater (Leica, Vienna, Austria) to provide electrical conductivity and prevent charge accumulation during the electron beam exposure. A secondary electron detector (ETD detector—Everhart–Thornley detector) was used in order to highlight the size and shape of the particles at an acceleration voltage of 5 kV and a spot size of 0.4 nA.

#### 2.4.6. Broadband Dielectric Spectroscopy (BDS)

The broadband dielectric spectroscopy measurements were undertaken on a broadband dielectric spectrometer (Novocontrol Technologies, Frankfurt, Germany). The dielectric parameters were recorded isothermally in a frequency window between 1 Hz and 1 kHz and at temperatures between −50 °C and 250 °C.

#### 2.4.7. Contact Angle Measurements

Static contact angle measurements were performed on a CAM–200 instrument (KSV Instruments, Helsinki, Finland) with a sessile drop profile analysis. The measurements were undertaken at room temperature by placing a 1 μL drop of liquid on the sample surface. Each contact angle value was a mean of five measurements taken on different surface sites.

## 3. Results and Discussion

### 3.1. Structural Characterization by FT-IR

**Figure 2:**
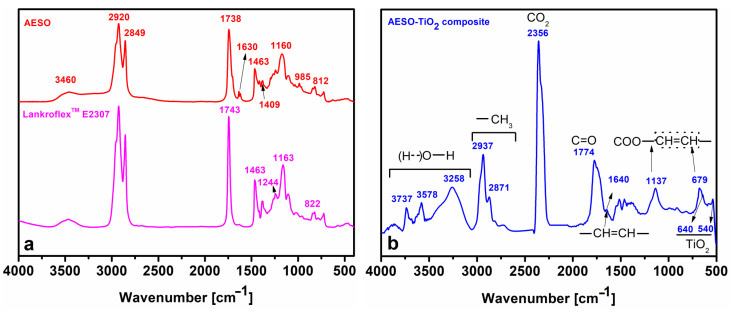
(**a**) The FT-IR spectra of the epoxidized soybean oil and AESO and (**b**) the FT-IR spectrum of the microcomposite.

Figure 2a shows the comparative FT-IR spectra of Lankroflex^TM^ E2307 and the AESO formulation. The signals at 822 cm^−1^ and 1244 cm^−1^ in the FT-IR spectrum of Lankroflex^TM^ E2307 demonstrate the presence of epoxy rings [33]. The FT-IR spectrum in AESO confirms the synthesis of its structure via the formation of peaks at 3460 cm^−1^, which corresponds to the –OH moiety of the epoxy ring opening and is associated with intermolecular polymeric hydrogen bonding [34]. The wider peak at 1738 cm^−1^ is due to the appearance of supplementary carbonyl (C = O) entities. The vibration of the –CH=CH_2_ in acrylate is depicted by the signals at 1409 cm^−1^, 985 cm^−1^, 812 cm^−1^ and 1630 cm^−1^ [34].

Figure S3 depicts the FT-IR spectrum of the pristine TiO_2_ microparticles. All peaks are specific to the anatase form of TiO_2_ and have been thoroughly explained in the literature [35]. The broad band in the range of 880–400 cm^−1^ is attributed to Ti–O stretching (420 cm^−1^) and Ti–O–Ti vibrations (620 cm^−1^; 537 cm^−1^). With the incorporation of the TiO_2_ microparticles into the composite (Figure 2b), compared to the TiO_2_ spectrum (Figure S3), the broad absorption band at 880−400 cm^−1^ almost disappears. Also, in the spectrum of AESO–TiO_2_, the peaks describing the Ti–O–Ti vibrations appear very weak and seem to have shifted towards higher wavelength values (640 cm^−1^; 540 cm^−1^). Moreover, the slightly detectable peak at 640 cm^−1^ is in conjunction with the peak at 679 cm^−1^ from the crosslink of the reactive diluent within the composite. This aspect demonstrates that the TiO_2_ microparticles were successfully incorporated into the AESO matrix during thermal curing [36].

Figure 2b shows the FT-IR spectrum of the microcomposite AESO–TiO_2_. Compared to the FT-IR spectra in Figure 2a, the complex FT-IR spectrum of the composite AESO–TiO_2_ contains more signals originating from the bulky TMPTMA structure and its curing with and within the AESO matrix [37]. According to the literature, the peaks in the range of 3000–3750 cm^−1^ and the one at 3258 cm^−1^ may correspond to the stretching vibrations of different hydrogen bonds between –OH entities. The peak at 1640 cm^−1^ corresponds to the terminal C=C bonds of TMPTMA, and the peak’s very low intensity indicates that these bonds are implicated in curing reactions. Due to their bulky nature, the TMPTMA molecules become sterically hindered, restricting mobility in the three-dimensional composite network and leaving some unreacted double bonds in the system [38]. The peak at 1774 cm^−1^ could be attributed to some new carbonyl structures [39]. The intense, sharp peak at 2356 cm^−1^ is due to the entrapment of CO_2_ at the surface during the creation of the composite [40].

### 3.2. Thermal and Morphological Characterization

**Figure 3:**
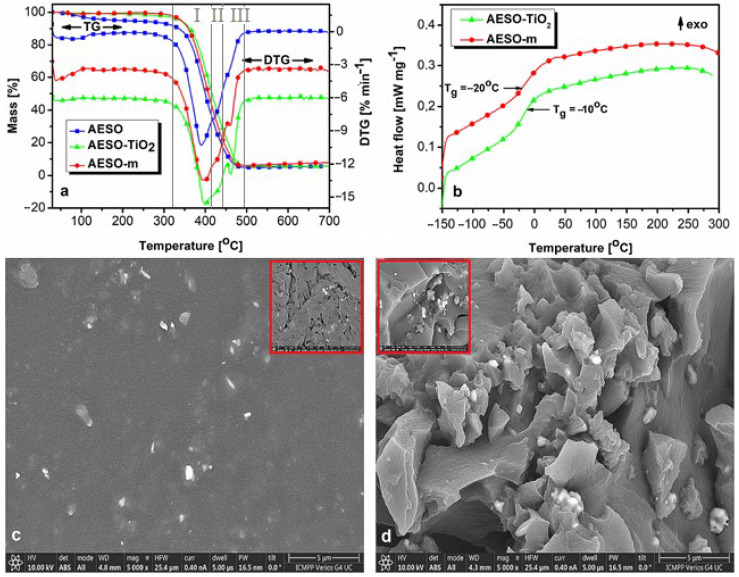
(**a**) Thermogravimetric curves, (**b**) DSC curves, (**c**) surface SEM micrographs of the cured matrix and (**d**) microcomposite. Scale marks at 5 µm and 1 µm (red box).

Figure 3a,b show the thermogravimetric curves, with their corresponding first derivative (DTG) curves, respectively. Table S1 in the Supplementary Materials lists the data extracted from the thermal analyses: the static heat resistant index (T_s_), calculated with Equation (1) [41], that is an overall value given by the parameters chosen as criteria to assess thermal stability (i.e., the temperatures at 5% (T_5%_) and 30% (T_30%_) mass loss); the temperature at a maximum rate of degradation (T_peak_); the mass loss (%) for each thermal degradation stage (M); the residual mass (%) at the end of the thermal degradation process (700 °C) (R) and the glass transition temperature (T_g_).

T_s_ = 0.49·[T_5%_ + 0.6·(T_30%_ − T_5%_)] (1)

The thermogravimetric curves (Figure 3a) show a significant increase in the thermal stability of the materials after curing, from T_s_ = 150 °C for AESO to T_s_ = 180 °C and 183 °C for the cured matrix (AESO–m) and composite (AESO–TiO_2_), respectively. The DTG curves indicate a thermal degradation profile in three overlapping stages, which is almost indiscernible in the TGA curves. The T_peak_ in the DTG curves shifts toward higher values with curing and addition of TiO_2_. Also, the mass loss (M) decreases for thermal degradation stages I and II and increases for stage III of AESO–m and AESO–TiO_2_ compared to AESO (Table S1). The three overlapping thermal degradation stages are due to an initial random chain scission, followed by almost simultaneous branching and crosslinking phenomena in AESO [42]. While the T_s_ and T_5%_ values of AESO–m and AESO–TiO_2_ are close, noteworthy differences arise at higher temperatures. During stage I of thermal degradation (320 °C–410 °C), where the mass loss is highest, AESO–TiO_2_ exhibits lower mass loss than AESO–m (48.89% vs. 57.14%), indicating improved thermal stability. This is also shown by the higher T_30%_ value for AESO–TiO_2_ compared to AESO–m (395 °C vs. 387 °C). Despite low TiO_2_ loading, physical filler–matrix interactions occur, which stiffen the matrix. Such a stiffening effect was also reported in other systems containing well-embedded nanostructures [43]. Also, the low filler load does not significantly increase the residual char, suggesting that TiO_2_ mainly influences AESO–TiO_2_ surface properties. This stiffening effect is also in good agreement with the SEM micrograph of the composite (Figure 3d), which shows a more compact texture compared to that of the cured matrix (Figure 3c). The more compact structure of AESO–TiO_2_ is also responsible for its higher T_g_ compared to that of AESO–m (Figure 3b). During stages II (410–440 °C) and III (440–490 °C) of the thermal degradation, where the crosslinks decompose, the low filler loading hinders chain packing in AESO–TiO_2_, as opposed to the stiffening effect in stage I. An increased free volume between chains leads to higher mass losses for AESO–TiO_2_ compared to those of AESO–m (Table S1). The DSC second heating curves of the studied materials (Figure 3b) show the presence of a single neat T_g_, demonstrating the formation of a fully thermally cured composite, a confirmation of good miscibility between the components and a uniform distribution of the TiO_2_ microparticles within the composite matrix. These aspects also led to an increase in the T_g_ of the composite from −20 °C (AESO–m) to −10 °C (AESO–TiO_2_), as confirmed by the SEM measurements in Figure 3c,d.

### 3.3. Wettability

**Figure 4:**
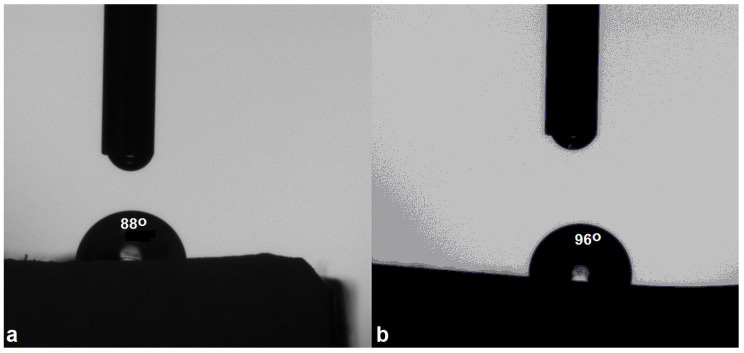
Water contact angle (**a**) of the cured epoxy matrix and (**b**) of the microcomposite.

Coatings are the largest application domain for epoxy resins. For epoxy materials to be used as anti-weathering, protective coatings and in microelectronics, it is crucial for them to possess hydrophobicity. The majority of surfaces, especially hygroscopic ones, are prone to damage due to liquid and water vapor absorption from the atmosphere, strongly affecting functional and mechanical characteristics, aesthetic features and biological durability. The application of hydrophobic coatings protects surfaces by limiting water ingress in both indoor and, especially, outdoor settings while also enhancing resistance against biological attacks [44]. Zou et al. [45] observed that nanofillers enhanced water sorption and decreased the T_g_ in epoxy materials, as opposed to microfillers, which had little contribution and exhibited higher T_g_ values. The AESO–TiO_2_ formulation can be applied in an oven via direct thermal curing on high-temperature-resistant surfaces, such as metals or ceramics. The water contact angle of the matrix and composite was measured. It was observed that, compared to the matrix’s contact angle value below 90°, the microcomposite possessed a contact angle value above 90° (Figure 4). This aspect implies that the incorporation of the TiO_2_ microparticles rendered the composite hydrophobic. The contact value of the matrix was 88°, indicating hydrophilic behavior. The microcomposite manifested hydrophobic characteristics, displaying a contact angle value of 96°. An explanation could reside in the microparticles’ mobility—as demonstrated by the increased permittivity in the dielectric studies discussed in the next section—allowing them to migrate in the polymer matrix, leading to a higher packing degree and the formation of a microstructured compact texture. This phenomenon not only increases surface hydrophobicity, but at the same time, generates good compatibility with the polymer matrix, as previously shown by the DSC and SEM results. Moreover, the anchoring of TiO_2_ microparticles may expose alkyl moieties at the solid−air interface that, together with the suitable morphology, also increase the contact angle value of the microcomposite [46].

### 3.4. BDS

**Figure 5:**
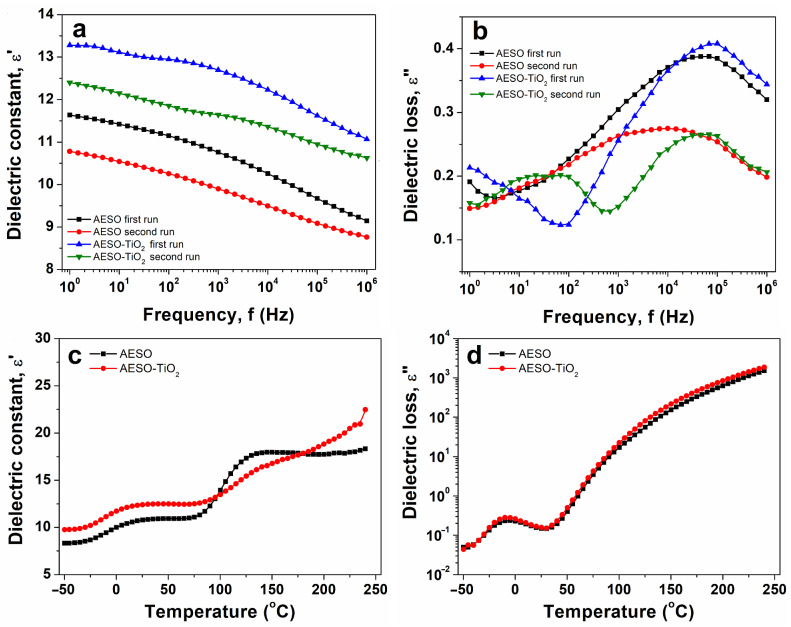
(**a**,**b**) The evolution of the dielectric constant and dielectric loss with frequency at room temperature. (**c**,**d**) The comparative evolution of the dielectric constant and dielectric loss with temperature at a frequency of 1 Hz.

Dielectric properties play a crucial role in assessing the use of epoxy resins as electrical insulators and are greatly influenced by their molecular structure [47]. The behaviors of the dielectric constant, ε′, and dielectric loss, ε″, parameters with frequency are presented in Figure 5. The isothermal plots are selected at room temperature. The ε′ parameter is related to the dipolar activity of a material. As observed in Figure 5a, ε′ decreases gradually with increasing frequency, as generally observed for polymer materials [48]. The magnitude of ε′ of the composite is higher than that of the cured matrix. The enhanced magnitude of ε′ for the composite may be due to the incorporation of the TiO_2_ filler in the matrix, which induces supplementary polarizable units [49]. The increased permittivity values of the matrix and the composite at lower frequencies may be associated either with the Maxwell–Wagner effect, when the alternating current and applied potential are in phase, or direct current conductivity, defined as the result of increased ion mobility, or both, as detailed in the literature [50,51]. The dielectric loss encompasses the dissipation energy required to align the polarizable units in the direction of an external electrical field. Similar to ε′(f) dependences, the magnitude of ε″ (Figure 5b) decreases considerably due to the vegetable oil entities.

The isochronal plots of ε′ and ε″ vs. the temperature of the studied materials are comparatively displayed in Figure 5c,d. The spectra are selected at 1 Hz. At temperatures lower than 0 °C, a clear relaxation process is retrieved as a step increase in the dielectric spectra of ε′—higher for the composite than for the matrix (Figure 5c)—and as a well-defined dielectric peak in the spectra of ε″ (Figure 5d). According to the DSC data, the relaxation process is connected with the glass transition temperature of the materials, found in both the ε′(T) and ε″(T) profiles of the two materials. At temperatures between 100 °C and 200 °C, a secondary step increase is detected in the ε′(T) profiles of both samples. This step increase may suggest a hindering or cleavage of physical bonds [52]. In the ε″(T) profiles of the structures, no relaxation process is detected between 100 °C and 200 °C.

It may be observed that the second step is slightly detected in the isochronal ε′(T) plot of the AESO–TiO_2_ composite, likely attributed to the incorporation of the TiO_2_ filler, which plays a key role in governing the composite’s dielectric properties.

The AESO–TiO₂ composite presents interesting dielectric properties that are promising for various electronic applications. As presented in the manuscript, the inclusion of the TiO_2_ filler into the AESO matrix increased the dielectric constant of the final product, while the dielectric loss parameter remained comparable between the matrix and the composite. The enhanced dielectric constant improved the ability of the material to store an electric charge, making it suitable for the fabrication of capacitors. On the other hand, the capability of the composite to maintain low dielectric losses minimized the energy dissipation, which may have reduced the signal degradation, making it suitable for use in sensors and electronic circuits [53,54,55].

## 4. Conclusions

This study describes the obtaining, structural characterization, morphology, wettability and the thermal and dielectric behavior of a green composite from a formulation of epoxidized and acrylated soybean oil containing 5% trimethylolpropane trimethacrylate as a reactive diluent, 2% TiO_2_ microparticles as a filler and 0.15 mL of triethylamine as a catalyst. The aim of this research was to obtain a microcomposite with a reduced initial matrix formulation viscosity and without the use of toxic curing agents. The material was obtained through thermal curing. The components showed excellent miscibility within the cured matrix. Compared to the pristine cured matrix, the 2% filler in the composite generated superior thermal stability through lower mass loss (48.89% vs. 57.14%) and increased degradation temperatures (395 °C vs. 387 °C). The T_g_ and contact angle also increased from −20 °C to −10 ° and from 88° to 96° C, respectively, rendering the microcomposite hydrophobic. The TiO_2_ filler also increased the dielectric constant of the composite, while the dielectric loss parameter remained comparable between the matrix and the composite. The enhanced dielectric constant improved the ability of the material to store electric charge. The capability of the composite to maintain low dielectric losses minimized energy dissipation. These results suggest that the microcomposite be recommended for protective coatings, capacitors, sensors and electronic circuits.

## Data Availability

The original contributions presented in this study are included in the article/Supplementary Material. Further inquiries can be directed to the corresponding authors.

## Supplementary Materials

The following supporting information can be downloaded at: https://www.mdpi.com/article/10.3390/polym16233363/s1, Text S1: Characterization of The Acrylated and Epoxidized Soybean Oil (AESO); Figure S1: The ^1^H–NMR spectrum of AESO; Figure S2: The first DSC heating curve of the AESO–TiO_2_ formulation; Figure S3: FT-IR spectrum of the pristine TiO_2_ microparticles; Table S1: Thermal analyses data. References [56,57] are cited in the Supplementary Materials.

## References

[1] Mora A.S., Tayouo R., Boutevin B., David G., Caillol S. (2018). Vanillin-Derived Amines For Bio-based Thermosets. Green Chem..

[2] Li R., Zhang P., Liu T., Muhunthan B., Xin J.N., Zhang J.W. (2018). Use of Hempseed-oil-derived Polyacid and Rosin-derived Anhydride Acid as Cocuring Agents for Epoxy Materials. ACS Sustain. Chem. Eng..

[3] Xin J.N., Li M., Li R., Wolcott M.P., Zhang J.W. (2016). Green Epoxy Resin System based on Lignin and Tung Oil and Its Application in Epoxy Asphalt. ACS Sustain. Chem. Eng..

[4] Ng F., Couture G., Philippe C., Boutevin B., Caillol S. (2017). Bio-based aromatic epoxy monomers for thermoset materials. Molecules.

[5] Roudsari G.M., Mohanty A.K., Misra M. (2014). Study of the Curing Kinetics of Epoxy Resins with Biobased Hardener and Epoxidized Soybean Oil. ACS Sustain. Chem. Eng..

[6] Maksym P., Tarnacka M., Dzienia A., Matuszek K., Chrobok A., Kaminski K., Paluch M. (2017). Enhanced Polymerization Rate and Conductivity of Ionic Liquid-Based Epoxy Resin. Macromolecules.

[7] Zhou J., Xu K., Xie M., Wu H., Hua Z., Wang Z. (2019). Two Strategies to Precisely Tune the Mechanical Properties of Plant Oil-derived Epoxy Resins. Compos. Part B Eng..

[8] Petrie E.M. (2006). Epoxy Adhesive Formulations.

[9] Visakh P.M., Rosu D. (2016). Photochemical Behavior of Multicomponent Polymeric-Based Materials.

[10] Liang X., Yin N., Liang S., Yang R., Liu S., Lu Y., Jiang L., Zhou Q., Jiang G., Faiola F. (2020). Bisphenol A and Several Derivatives Exert Neural Toxicity In Human Neuron-like Cells by Decreasing Neurite Length. Food Chem. Toxicol..

[11] Qiu W., Zhan H., Hu J., Zhang T., Xu H., Wong M., Xu B., Zheng C. (2019). The Occurrence, Potential Toxicity, and Toxicity Mechanism of Bisphenol S, a Substitute of Bisphenol A: A Critical Review of Recent Progress. Ecotoxicol. Environ. Saf..

[12] Godiya C.B., Park B.J. (2022). Removal of bisphenol A from Wastewater by Physical, Chemical and Biological Remediation Techniques. A Review. Environ. Chem. Lett..

[13] Di Mauro C., Tran T.N., Mija A. (2021). One-pot Terpolymerization Synthesis of High Carbon Biocontent Recyclable Epoxy Thermosets and Their Composites with Flax Woven Fibers. ACS Sustain. Chem. Eng..

[14] Mustata F., Tudorachi N., Bicu I. (2016). Curing Kinetics, Thermal and Morphological Characterization of the Biobased Thermosets from Epoxy Resin/Epoxidized Hemp Oil. J. Anal. Appl. Pyrolysis.

[15] Rosu D., Mustata F., Tudorachi N., Musteata V.E., Rosu L., Varganici C.D. (2015). Novel Bio-based Flexible Epoxy Resin from Diglycidyl Ether of Bisphenol A Cured with Castor Oil Maleate. RSC Adv..

[16] Mustata F., Tudorachi N., Bicu I. (2014). The Curing Reaction of Epoxidized Methyl Esters of Corn Oil with Diels-Alder Adducts of Resin Acids. The Kinetic Study and Thermal Characterization of Crosslinked Products. J. Anal. Appl. Pyrolysis.

[17] Worzakowska M. (2007). The Kinetic Study of the Curing Reaction of Mono- and Diepoxides Obtained During the Reaction of Divinylbenzene and Hydrogen Peroxide with Acid Anhydrides. Polymer.

[18] Yang X., Wang C., Li S., Huang K., Li M., Mao W., Cao S., Xia J. (2017). Study on the Synthesis of Bio-based Epoxy Curing Agent Derived from Myrcene and Castor Oil and the Properties of the Cured Products. RSC Adv..

[19] Jagtap A.R., More A. (2022). Developments in Reactive Diluents: A Review. Polym. Bull..

[20] Chen L., Zhang Y., Chen Z., Dong Y., Jiang Y., Hua J., Liu Y., Osman A.I., Farghali M., Huang L. (2024). Biomaterials Technology and Policies in the Building Sector: A Review. Environ. Chem. Lett..

[21] Anastas P., Eghbali N. (2010). Green Chemistry: Principles and Practice. Chem. Soc. Rev..

[22] Chong K.L., Lai J.C., Rahman R.A., Adrus N., Al-Saffar Z.H. (2021). Self-healable Biobased Epoxy Resin from Epoxidized Palm Oil. Chem. Eng. Trans..

[23] Zhang C., Garrison T.F., Madbouly S.A., Kessler M.R. (2017). Recent Advances in Vegetable Oil-based Polymers and Their Composites. Prog. Polym. Sci..

[24] Kadam A., Pawar M., Yemul O., Thamke V., Kodam K. (2015). Biodegradable Biobased Epoxy Resin from Karanja Oil. Polymer.

[25] Tsujimoto T., Takeshita K., Uyama H. (2016). Bio-based Epoxy Resins from Epoxidized Plant Oils and Their Shape Memory Behaviors. J. Am. Oil Chem. Soc..

[26] Varganici C.D., Rosu L., Rosu D., Mustata F., Rusu T. (2021). Sustainable Wood Coatings Made of Epoxidized Vegetable Oils for Ultraviolet Protection. Environ. Chem. Lett..

[27] Rosu L., Mustata F., Rosu D., Varganici C.D., Rosca I., Rusu T. (2021). Bio–based Coatings from Epoxy Resins Crosslinked with a Rosin Acid Derivative for Wood Thermal and Anti-fungal Protection. Prog. Org. Coat..

[28] Salarbashi D., Bazeli J., Tafaghodi M. (2019). Environment-friendly Green Composites based on Soluble Soybean Polysaccharide: A Review. Int. J. Biol. Macromol..

[29] Pin J.M., Sbirrazzuoli N., Mija A. (2015). From Epoxidized Linseed Oil to Bioresin: An Overall Approach of Epoxy/Anhydride Cross-linking. ChemSusChem.

[30] Mustata F., Tudorachi N. (2016). Thermosets Based on Castor Oil Modified with Diels-Alder Adduct of Levopimaric Acid and Diglycidyl Ether of Bisphenol A. The Kinetic Analysis of the Curing Reactions and Thermal Behavior of the Cured Products. Compos. Part B Eng..

[31] Zia K.M., Noreen A., Zuber M., Tabasum S., Mujahid M. (2016). Recent Developments and Future Prospects on Bio-based Polyesters Derived from Renewable Resources: A Review. Int. J. Biol. Macromol..

[32] Nawaz H., Umar M., Maryam R., Nawaz I., Razzaq H., Malik T., Liu X. (2022). Polymer Nanocomposites Based on TiO_2_ as a Reinforcing Agent: An Overview. Adv. Eng. Mater..

[33] Rana A., Evitts R.W. (2015). Synthesis and Characterization of Acrylated Epoxidized Flaxseed Oil for Biopolymeric Applications. Int. Polym. Proc..

[34] Balaban A.T., Banciu M., Pogany I. (1983). Applications of Physical Methods in Organic Chemistry.

[35] Praveen P., Viruthagiri G., Mugundan S., Shanmugam N. (2014). Structural, Optical and Morphological Analyses of Pristine Titanium Di-oxide Nanoparticles—Synthesized via Sol–Gel Route. Spectrochim. Acta A.

[36] Shafaamri A., Cheng C.H., Ma I.A.W., Baig S.B., Kasi R., Subramaniam R., Balakrishnan V. (2020). Effects of TiO_2_ Nanoparticles on the Overall Performance and Corrosion Protection Ability of Neat Epoxy and PDMS Modified Epoxy Coating Systems. Front. Mater..

[37] Lai W., Li X., Liu H., Han L., Zhao Y., Li X. (2014). Interfacial Polycondensation Synthesis of Optically Sensitive Polyurea Microcapsule. J. Chem..

[38] Gunewardena A., Gilbert M. (2008). Peroxide Crosslinking of Rigid Poly(vinyl chloride). J. Vinyl Addit. Technol..

[39] Silverstein R.M., Webster F.S., Kiemle D.J. (2005). Spectrometric Identification of Organic Compounds.

[40] Varganici C.-D., Rosu L., Rosu D., Rosca I., Ignat M.-E., Ignat L. (2024). Surface Degradation of DGEBA Epoxy Resins Cured with Structurally Different Amine Hardeners: Effects of UV Radiation. Polymers.

[41] Mustata F., Rosu D., Varganici C.D., Rosu L., Rosca I., Tudorachi N. (2022). Assessing the Thermal and Fungal Behavior of Eco–friendly Epoxy Thermosets Derived from Vegetable Oils for Wood Protective Coatings. Prog. Org. Coat..

[42] Behera D., Banthia A.K. (2008). Synthesis, Characterization, and Kinetics Study of Thermal Decomposition of Epoxidized Soybean Oil Acrylate. J. Appl. Polym. Sci..

[43] Bifulco A., Avolio R., Lehner S., Errico M.E., Clayden N.J., Pauer R., Gaan S., Malucelli G., Aronne A., Imparato C. (2023). In Situ P-Modified Hybrid Silica–Epoxy Nanocomposites via a Green Hydrolytic Sol–Gel Route for Flame-Retardant Applications. ACS Appl. Nano Mater..

[44] Fink J.K. (2013). Epoxy Resins. Reactive Polymers Fundamentals and Applications. A Concise Guide to Industrial Polymers.

[45] Zou C., Fothergill J.C., Rowe S. (2008). The Effect of Water Absorption on the Dielectric Properties of Epoxy Nanocomposites. IEEE Trans. Dielectr. Electr. Insul..

[46] Passaro J., Bifulco A., Calabrese E., Imparato C., Raimondo M., Pantani R., Aronne A., Guadagno L. (2023). Hybrid Hemp Particles as Functional Fillers for the Manufacturing of Hydrophobic and Anti-icing Epoxy Composite Coatings. ACS Omega.

[47] Li C., Fan H., Aziz T., Bittencourt C., Wu L., Wang D.Y., Dubios P. (2018). Biobased Epoxy Resin with Low Electrical Permissivity and Flame Retardancy: From Environmental Friendly High-Throughput Synthesis to Properties. ACS Sustain. Chem. Eng..

[48] Kremer F., Schönhals A. (2003). Broadband Dielectric Spectroscopy.

[49] Chen S., Yao K., Tay F.E.H., Liow C.L. (2007). Ferroelectric Poly (vinylidene fluoride) Thin films on Si substrate with the β phase promoted by hydrated magnesium nitrate. J. Appl. Phys..

[50] Saad A.L.G., Hassan A.M., Youssif M.A., Ahmed M.G.M. (1997). Studies of Electrical Properties of Some Fire-retarding Poly (vinyl chloride) Compositions. J. Appl. Polym. Sci..

[51] Abd–El–Messieh S., Naguib H. (2005). Electrical conductivity and dielectric behavior of some poly(alkyl methacrylate) s/polyvinylpyrrolidone blends. Polym.-Plast. Technol. Eng..

[52] Zhang D., Runt J. (2004). Segmental dynamics and ionic conduction in poly (vinyl methyl ether)–lithium perchlorate complexes. J. Phys. Chem. B.

[53] Shubha A., Manohara S.R., Angadi B. (2022). Influence of TiO_2_ Nanoparticles on Structural, Optical, Dielectric and Electrical Properties of Bio–compatible PEOX–PVP–TiO_2_ Nanocomposites. Polym. Bull..

[54] Ravikiran Y.T., Gangu Naidu C.H., Prashantkumar M., Alla S.K., Ramana C.H.V.V. (2023). 14—Polymer Blend Nanocomposites for Capacitor Applications. Polymer Blend Nanocomposites for Energy Storage Applications, Micro and Nano Technologies.

[55] Sun Z., Wong R., Yu M., Li J., Zhang M., Mele L., Hah J., Kathaperumal M., Wong C.-P. (2022). Nanocomposites for Future Electronics Device Packaging: A Fundamental Study of Interfacial Connecting Mechanisms and Optimal Conditions of Silane Coupling Agents for Polydopamine–Graphene Fillers in Epoxy Polymers. Chem. Eng. J..

[56] Campanella A., La Scala J.J., Wool R.P. (2011). Fatty Acid–Based Comonomers as Styrene Replacements in Soybean and Castor Oil-Based Thermosetting Polymers. J. Appl. Polym. Sci..

[57] Saithai P., Lecomte J., Dubreucq E., Vanrattanakul V. (2013). Effects of different epoxidation methods of soybean oil on the characteristics of acrylated epoxidized soybean oil–co–poly(methyl methacrylate) copolymer. Express Polym. Lett..

