# Supplementary material for: From Fossil to Bio-Based AESO–TiO2 Microcomposite for Engineering Applications
Source: Polymers (Basel). 2024 Nov 29;16(23):3363. doi: 10.3390/polym16233363 (PMC11644145; doi:10.3390/polym16233363)
Supplement: Supplementary file 1 [file polymers-16-03363-s001.zip › polymers-3325907-supplementary.pdf]

Supplementary Materials

From fossil to bio–based AESO–TiO_2 microcomposite for engineering applications

Cristian–Dragos Varganici*, Liliana Rosu, Dan Rosu, Mihai Asandulesa

“Petru Poni” Institute of Macromolecular Chemistry, 41A Gr. Ghica–Voda Alley, 700487 Iasi, Romania

* Corresponding author. E-mail: varganici.cristian@icmpp.ro

Text S1: Characterization of the acrylated and epoxidized soybean oil (AESO)

The presence of the acrylate group in AESO was demonstrated via the ^{1}H–NMR technique. Figure S1 depicts the ^{1}H–NMR spectrum of AESO. The signal peaks at 5.7–6.2 ppm are consistent with acrylate ester entities. The signal peaks from 5.1–5.3 ppm and 4–4.5 ppm denote the –CH_2–CH–CH_2– glycerol methine and glycerol methylene protons, respectively, from the triglyceride backbone [1]. The weak signals in the range 3.2–4 ppm correspond to each hydroxyl group created for every acrylate moiety bonded to a triglyceride. The low intensity peaks in the range 2.8–3.2 ppm describe the protons of the epoxide ring owed to incomplete acrylation. A complete acrylation reaction of epoxidized soybean oil has not yet been reported, due to the bulkiness of the triglyceride molecule [2]. The following peaks are also attributed: –$(CH_2)n$– at 1.1–1.4 ppm, terminal –CH_3 at 0.9–1.1 ppm, α–CH_2–(C=O)–O– at 2.2–2.4 ppm and β–CH_2–(C=O)–O– at 1.6–1.8 ppm [2]. In order to calculate the number of acrylate groups per molecule AESO or acrylation degree the peak of terminal –CH_3 groups (0.9–1.1 ppm) was used as internal standard. The acrylation degree was estimated from the ratio of peak areas $A_{5.7–6.2\ ppm}/A_{0.9–1.1\ ppm}$ and found to be 2.42. This is in good agreement with literature data, according to which the acrylation degree for the commercial AESO Vikoflex© 7170, for example, is 2.76 [2].

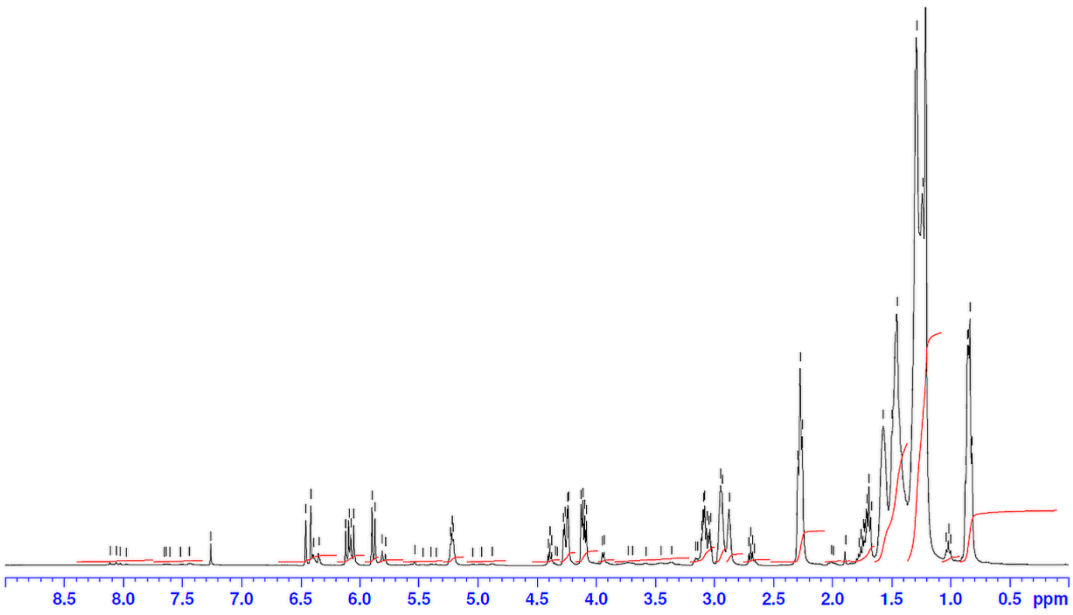

Figure S1. The ^1H–NMR spectrum of AESO

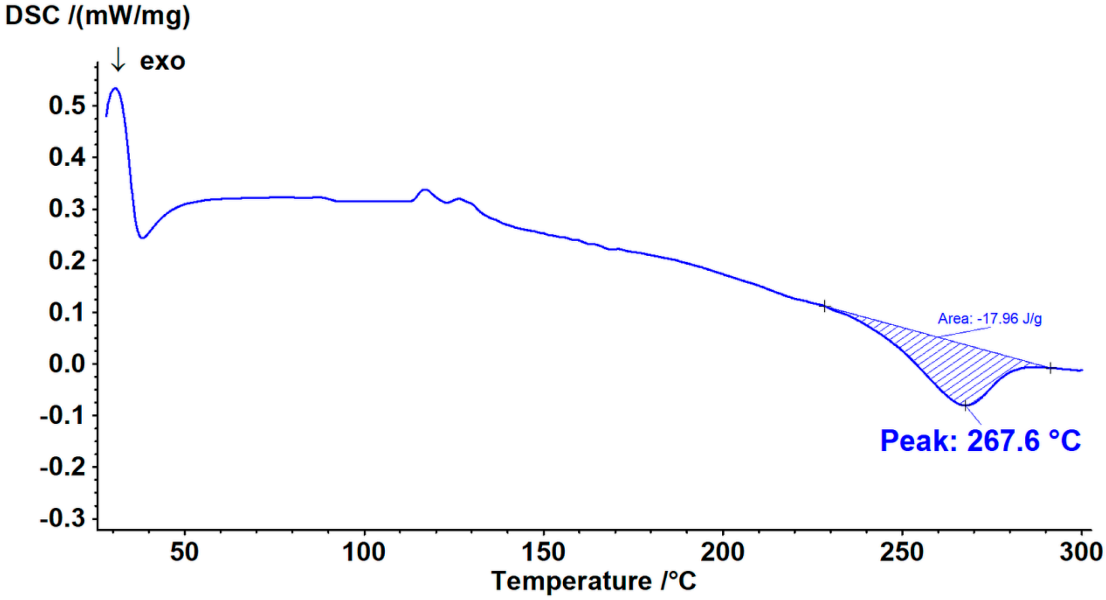

Figure S2. The first DSC heating curve of AESO –TiO_2 formulation.

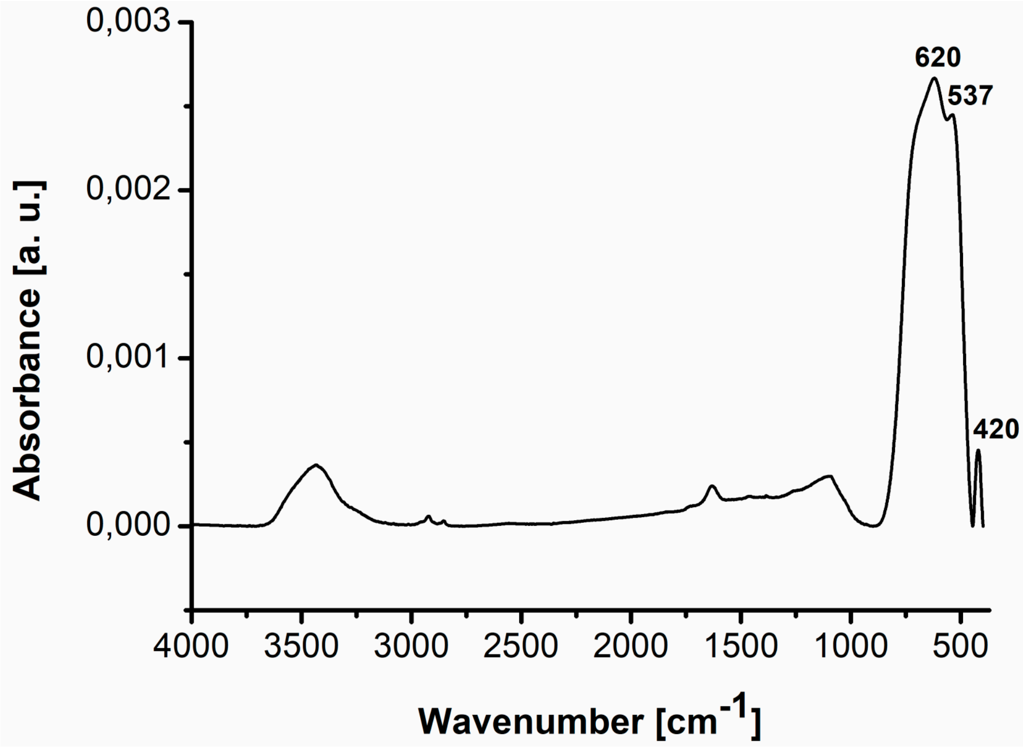

Figure S3. FT–IR spectrum of the pristine TiO_2 microparticles.

Table S1. Thermal analyses data.

Sample	Stage	T_s (°C)	$T_{5\%}$ (°C)	T_{peak} (°C)	$T_{30\%}$ (°C)	M (%)	R (%)	T_g (°C)
	I			390		64.31		
**AESO**	II	150	197	420	380	17.83	5.46	–
	III			454		12.35		
	I			393		57.14		
**AESO–m**	II	180	340	426	387	19.85	7.49	–20
	III			460		14.71		
	I			400		48.89		
**AESO–TiO_2**	II	183	342	430	395	28.17	5.52	–10
	III			462		16.49		

T_s – static heat resistant index; $T_{5\%}$ – temperature at 5% mass loss; T_{peak} – temperature of maximum rate of decomposition; $T_{30\%}$ – temperature at 30% mass loss; M – percentage of mass loss for each stage; R – percentage of mass residue remained at 700 °C; T_g – glass transition temperature

References

1. Campanella, A; La Scala, JJ; Wool RP. (2011) Fatty Acid–Based Comonomers as Styrene Replacements in Soybean and Castor Oil-Based Thermosetting Polymers. *J. Appl. Polym. Sci.* **2011**, *119*, 1000–1010. DOI: 10.1002/app.32810
2. Saithai, P; Lecomte, J; Dubreucq, E; Vanrattanakul, V. Effects of different epoxidation methods of soybean oil on the characteristics of acrylated epoxidized soybean oil–co–poly(methyl methacrylate) copolymer. *Express Polym. Lett.* **2013**, *7*, 910–924. DOI: 10.3144/expresspolymlett.2013.89
